# Comparing ChatGPT and Dental Students' Performance in an Introduction to Dental Anatomy Examination: A Cross-Sectional Study

**DOI:** 10.1055/s-0045-1808254

**Published:** 2025-05-13

**Authors:** Rizwan Ullah, Muhammad Saad Shaikh, Nazish Shahani, Mohid Abrar Lone, Muhammad Amber Fareed, Muhammad Sohail Zafar

**Affiliations:** 1 Department of Oral Biology, Sindh Institute of Oral Health Sciences, Jinnah Sindh Medical University, Karachi, Pakistan; 2 Department of Oral Pathology, Sindh Institute of Oral Health Sciences, Jinnah Sindh Medical University, Karachi, Pakistan; 3 Clinical Sciences Department, College of Dentistry, Ajman University, Ajman, United Arab Emirates; 4 Centre of Medical and Bio-allied Health Sciences Research, Ajman University, Ajman, United Arab Emirates; 5 School of Dentistry, University of Jordan, Amman, Jordan

**Keywords:** dental anatomy, artificial intelligence, dental education, ChatGPT, tooth morphology

## Abstract

**Objectives:**

This article compares the knowledge and interpretation ability of Chat Generative Pre-Trained Transformer (ChatGPT), with undergraduate dental students by administering a dental anatomy multiple-choice question-based examination.

**Materials and Methods:**

This analytical cross-sectional study determined ChatGPT's justification for each response to evaluate its suitability as an e-learning tool. The frequency and percentage of students and ChatGPT were calculated to obtain the correct answers for a multiple-choice examination.

**Statistical Analysis:**

The data analysis was performed through Statistical Package for Social Sciences (SPSS) by IBM (Version 20) and Microsoft Excel by Microsoft Corporation. The frequency and percentage of students and the ChatGPT were calculated for the correct answers. The *p* -value of the Shapiro–Wilk test was 0.001, therefore, the Kolmogorov test was applied to check the hypothesis for the distribution of the average ChatGPT explanation score given by the experts.

**Results:**

The results revealed that students performed better in the introductory dental anatomy examination. The average score of students was 74.28%, while that of ChatGPT was 60%. A good agreement was observed between the experts regarding the grading of the explanation.

**Conclusion:**

ChatGPT possesses a foundational understanding of basic dental anatomy, sufficient to achieve a passing grade on an undergraduate examination, its performance exhibits limitations in accuracy and reliability, therefore, it cannot be recommended as a sole learning resource.

## Introduction

The emphasis on e-learning and hybrid learning has significantly transformed the educational system in recent years. This disruption became prominent during the coronavirus disease 2019 pandemic, when higher education institutions successfully implemented information and communication technologies for teaching and assessment. 1 2 3 4 The second disruptive phase in education begins with the introduction of artificial intelligence (AI). Like other fields of education, AI has greatly transformed the clinical and educational aspects of dentistry 5 and has been advocated for a promising potential for health care education. 6 A few examples of the applications of AI in dental education include designing removable partial dentures using a game-based approach, 7 the development of multiple-choice questions (MCQs) using generative AI with a final evaluation by an expert, 8 and an AI cone-beam computed tomography system for generating a report of each tooth. 9

Open AI, an initiative that applies AI, has created an open-sourced cutting-edge Chat Generative Pre-Trained Transformer (ChatGPT), which is likely to transform significant changes in the field of education. 10 ChatGPT processes and produces natural language content by using deep learning type of machine learning. The dialog format of the ChatGPT interface provides detailed responses to queries, and subsequent questions acknowledges mistakes and rejects unsuitable questions or prompts. 11 12 13 14 15 Consequently, ChatGPT can produce high-quality texts that are difficult to differentiate from human writing. Furthermore, it can also carry out challenging human-like tasks such as writing novels, poems, and programming codes. 16 Additionally, ChatGPT can aid in automated scoring and help teachers and students by offering precise and fast access to information, which may help students solve quizzes, flashcards, and writing assignments without learning the desired knowledge. 13 14

Numerous studies addressing the use of ChatGPT in education and assessment have recently been published. Examples include the ability of ChatGPT to solve and explain questions from the practice sections of the United States Medical Licensing Examination, 17 Chinese National Medical Licensing Examination, 18 ChatGPT's ability to pass an introductory physics examination, 19 and its application in school science education. 20 In addition to that in the clinical settings, AI is being used for facial-based population screening for genetic diseases. Therefore, the educational community has raised such concerns because of its potential disruption in the educational system. For instance, ChatGPT may help students with writing assignments and essays 21 that cannot be easily detected by the majority of plagiarism-checking software, 22 help students with solving online assessments and assignments, 11 23 and scientific manuscripts writing. 24 25 In dentistry, the manuscripts examines the use of ChatGPT 3.5 and 4 in answering the dental board-style questions, 26 but the earlier model of ChatGPT did not perform sufficiently well in answering the questions. ChatGPT performance in periodontics in service examination was shown to have high proficiency with ChatGPT 4 as compared to 3.5. 27 Another periodontology in service examination comparing efficiency of three AI platforms suggest that ChatGPT 4 performance was superior to the earlier version of ChatGPT and Google Gemini. 28 Currently, there are limited evidence about the performance and potential of ChatGPT in solving MCQs test of basic dental anatomy. In this study, we aimed to evaluate ChatGPT's performance on a MCQs test of basic dental anatomy and compare it to the scores of students who took a similar test. In addition, the present study looked at and rated ChatGPT's justification for each response to further evaluate its suitability as an e-learning tool. To the best of our knowledge, our study is among the few that provides valuable insights into the potential of AI, specifically large language models like ChatGPT, as a supplementary tool in dental anatomy education. By evaluating its performance against dental students, we can begin to explore how AI can be integrated to enhance learning and assessment.

## Materials and Methods

This study was formulated using the Strengthening the Reporting of Observational Studies in Epidemiology (STROBE) Statement: Guidelines for reporting observational studies. 29

### Sampling Method and Participants

This cross-sectional analytical study was conducted to investigate the performance of ChatGPT 3.5 in comparison with undergraduate dental students and the ability of ChatGPT to provide a correct explanation of those questions. This study was approved by the Institutional Review Board of Jinnah Sindh Medical University, Karachi, Pakistan (JSMU/IRB/2023/713) and was conducted by administering a formative assessment test among undergraduate dental students and ChatGPT, which was considered one of the examinees.

The study covers the entire class without the need for a sampling method, therefore, the total number of students in the class, that is, 50, were the sample size of this study. This study was performed from May to June 2023. Study participants were the first-year undergraduate students of the dentistry study program at the Sindh Institute of Oral Health Sciences, Jinnah Sindh Medical University, Karachi, Pakistan. They were strongly encouraged to participate in the test but their participation remained voluntary. The name and other personal information of the study participants were protected. Students were informed about the study and signed a consent form.

### Eligibility Criteria

#### Inclusion Criteria

1. We included 25 questions from the Department of Oral Biology question bank covering the introductory concepts of dental anatomy.
2. All the consenting students of first professional Bachelor of Dental Surgery.

#### Exclusion Criteria

1. We excluded any question that includes an image and tables. This will be done because ChatGPT 3.5 only accepts narrative text inputs rather than complex tabular text, charts, and images.
2. Students not giving consent or absent on the day of examination.

### Structure of the Exam

We included 25 MCQs with one best answer out of four options from the Department of Oral Biology question bank ( Supplementary File , available in the online version), covering the introductory concepts of dental anatomy, including terminologies, related to human dentition, structure of tooth, surfaces of teeth, and landmarks present on human dentition. The questions were prepared and later reviewed by faculty members of the department for the content validation; therefore, there is a minimal chance that these questions are present on the Internet and indexed in search engines such as Google and Yahoo. The Cronbach's alpha value of 0.776 indicates good reliability, indicating that the 25 items in the test are consistently measuring the same construct.

The students were allotted a total of 25 minutes for completing the examination. The examination was conducted in one of the lecture halls of the university and it was a paper-based examination administered and invigilated by the Faculty of the Department of Oral Biology.

Individual question papers along with the bubble answer sheets were administered to the students. The bubble answer sheets were manually checked by the investigators on the same day and the demographic information and the total score of each student were entered into an Excel sheet for further analysis.

### Grading of the Students

**Fig. 1:**
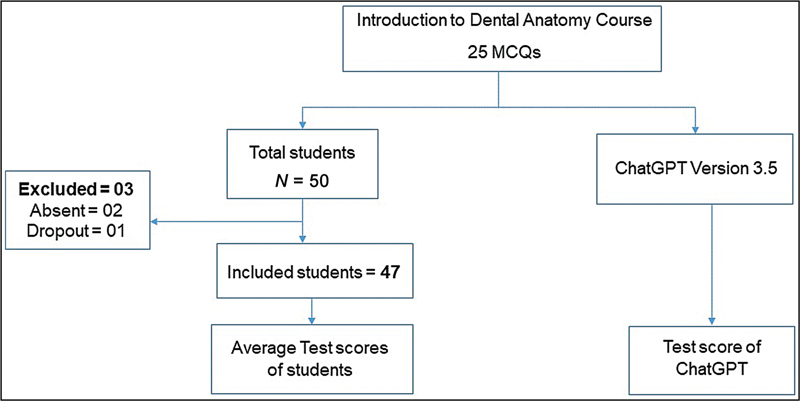
Summary of the study design and participants.

The 25 MCQs test was administered to undergraduate dental students on May 8, 2023. For each correct answer, one mark was awarded and zero was given for the incorrect answer. The average scores of the students on the test were then compared with the ChatGPT. Students who were absent on the test day, failed to provide written consent, and were previously enrolled in the dentistry program were excluded from the study. Fig. 1 provides a summary of the study design and participants.

### Question Input into ChatGPT and Grading

The same questions that were given to the students were entered into the ChatGPT version 3.5 on the same day by giving a prompt for the correct answer ( Supplementary File , available in the online version). There was only single attempt for each question and the time between each question/query was around 1 minute. The previous inputs were cleared before the next question, and each question was entered into a new chat session to avoid memory retention bias. When the answer given by the ChatGPT matched the question bank answer key, one mark was assigned. Like the students, the scores of the ChatGPT were entered into the Excel sheet for further analysis.

### Grading of the ChatGPT Explanation

Two subject specialists (R.U. and M.S.S.) in oral biology, with a Master's degree and more than 7 years of teaching experience, independently scored the explanation of each answer given by ChatGPT 3.5 on a scale from 0 to 10. The evaluation criteria used for giving score were subjective as the assessors were subject experts in oral biology. The interrater agreement between the authors on grading was calculated via Spearman's rank correlation ( Supplementary File , available in the online version).

### Statistical Analysis

The data analysis was performed through Statistical Package for Social Sciences (SPSS) by IBM (Version 20) and Microsoft Excel by Microsoft Corporation. The frequency and percentage of students and the ChatGPT were calculated for the correct answers. For each examinee, including ChatGPT for each correct answer, one mark was awarded and zero marks were given for incorrect answers. The explanation of the ChatGPT was independently examined by two subject experts (R.U. and M.S.S.) and graded on a score of 1 to 10. The *p* -value of Shapiro–Wilk test was 0.001, which shows that the data is not normal. Therefore, a nonparametric one-sample Kolmogorov test was applied to check the hypothesis for the distribution of the average score given by the expert to ChatGPT. The *p* -value was 0.004, which demonstrated a significant difference in the explanation score of ChatGPT from question 1 to question 25 ( Supplementary Tables A1 and A2 , available in the online version).

**Table 1:** The overall frequency and percentage of students' test scores and ChatGPT scores

Students ( *n* = 47)		ChatGPT	
**Frequency**	Percentage	Frequency	Percentage
18.57	74.28	15	60
Minimum correct = 12, maximum correct = 23			
Less than equal 15 correct is 5 students			

**Table 2:** The questions attempted incorrectly by the ChatGPT versus the percentage of students who attempted correctly

ChatGPT wrong answer	Percentage of students' correct answer
Q5	98
Q6	100
Q7	72
Q8	91
Q9	91
Q10	87
Q11	38
Q14	89
Q23	49
Q25	28

## Results

The total number of students who participated in this study was 47 out of the batch of 50 students in the first professional dentistry. From a total of 47 students, 14 were males and 33 were females. Regarding the completion rate, all the students who appeared in the examination completed the test paper.

The students performed better in the dental anatomy exam, with an average score of 18.57 (74.28%), as compared to ChatGPT, which scored 15 marks (60%). Out of the 25 questions, the minimum score of the students was 12, and the maximum score was 23. It is worth noting that as compared to the ChatGPT score, that is, 15, only 5 students scored 15 or less than 15 marks as shown in Table 1 .

The students scored better on the questions attempted wrong by the ChatGPT ( *n* = 10). Table 2 explains the overall percentage of students' correct answers to the questions attempted wrong by the ChatGPT.

In Table 3 , we also compared the questions in which the students scored less than 50% with the ChatGPT score. Out of six questions where students' scores were less than 50%, half of the questions were also attempted incorrectly by the ChatGPT.

**Table 3:** Questions in which the overall students' score is less than 50% compared to the ChatGPT score

Question number	Percentage of students' correct answers (less than 50%)	ChatGPT score
Q11	38	0
Q12	40	1
Q18	43	1
Q22	21	1
Q23	49	0
Q25	28	0

To evaluate the suitability of ChatGPT as an e-learning resource, grading was given to the explanation of the questions. Of the 25 questions, 14 had an average explanation score of less than 5. A good agreement was observed between the grading by the experts based on Spearman's rank correlation test *r* = 0.942, which is significant ( Supplementary Table A3 , available in the online version). Table 4 provides an overview of the questions administered to the ChatGPT with an average explanation score of less than 5. Questions 17, 19, 20, and 21 were correctly answered by the ChatGPT but received an average score of less than 5.

**Table 4:** Questions numbers that were administered to ChatGPT with an average explanation score of less than 5

Question number	Average score of explanation (max score 10)	Correct or wrong answer
Q5	4	0
Q6	1	0
Q7	1.5	0
Q8	0	0
Q9	0	0
Q10	0	0
Q11	0	0
Q14	0	0
Q17	0	1
Q19	4	1
Q20	2	1
Q21	1.5	1
Q23	0	0
Q25	0	0

**Fig. 2:**
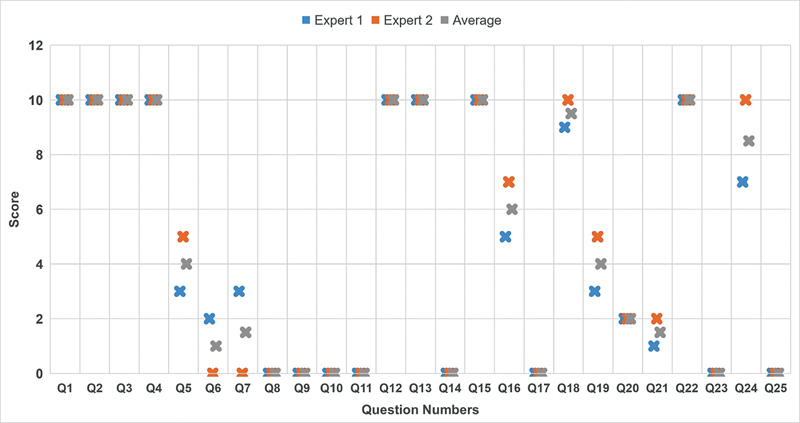
Box plot revealing a detailed overview of the explanation scores of all the questions provided by each expert and overall score.

Fig. 2 shows a box plot revealing a detailed overview of the scores provided by Expert 1 and Expert 2 and the average scores of all the questions.

## Discussion

The present study investigated the performance of ChatGPT for solving basic dental anatomy MCQs and compared it to the scores of students. In addition, ChatGPT's justification for each response was evaluated for its suitability as a potential e-learning tool for teaching dental subjects. The findings of this study offer a new perspective on the use of ChatGPT for exam preparation and as an e-learning resource. In our study, the percentage score for the ChatGPT was 60%. This finding is in agreement with the study by Gilson et al, where ChatGPT scored high percentages, 64.4 and 57.8%, in the National Board of Medical Examiners questions, step 1 and 2 exams, respectively. 17

In another study, the ChatGPT score on an introductory physics MCQ exam was also 60%. 19 The score of the ChatGPT was 15 out of 25 (60%), which is less than the average score of dental students 18.5 (74.2%). The ChatGPT score was sufficient to pass the basic dental anatomy examination (12.5; 50 %). In another study that explored ChatGPT's ability to pass the parasitology exam compared to the medical students, the results revealed that the ChatGPT score was 60.8% as compared to the medical students 90.8%. 30 The knowledge-based assessment in the dental education setting is largely face-to-face under direct vigilance, therefore, ChatGPT is not a direct threat for assessment. However, these findings may have important implications in the future for dental education and indicate the need for assessment to review and improve teaching curriculum design, exam policies, and assessments especially if any pandemic emerge again. 31

There were 15 questions out of 25 questions in which the explanation provided by ChatGPT received an average grading of less than 5 on a scale of 10 ( Table 4 ). The possible explanation for these low scores may be attributed to the limited ChatGPT data for certain specialties and difficulty in understanding the input or prompts. 14 32 As a result of this, some of the explanations make no sense or are partially correct explanations, one of the explanations had a one-word answer, and in another question, ChatGPT failed to provide any explanation even after multiple attempts ( Supplementary Figs. A1 – A4 , available in the online version). The possible explanations for this phenomenon are that ChatGPT is a general-purpose AI program and is not specifically trained in the medical and dental literature. The information used by ChatGPT is retrieved from the freely available literature, which is sometimes not sufficient or correct, and ChatGPT also uses some nonmedical and old resources. 32 33 The clinical images and diagrams are an important part of the dental anatomy, but due to the inability of the ChatGPT to process images and diagrams, this study did not include this type of question. 31 Another study by Huh provided the unique reasons for ChatGPT's lower performance in the parasitology exam by explaining that some data is unique to a specific country or region, which is not widely available online due to the availability of data in certain languages such as Korean. Similarly, ChatGPT has difficulty interpreting medical figures, graphs, and tables. 30 Therefore, ChatGPT provides some targeted and relevant information, which may not be sufficient to completely substitute the role of the subject matter expert, hence we cannot solely rely on ChatGPT as a standalone e-learning resource for dental anatomy education. Consequently, the teachers and students using ChatGPT and other similar AI platforms for teaching and in the clinical settings for diagnosis should understand the ethical and legal issues, bias, misuse, and privacy concerns, its limitations, and benefits, and be able to critically analyze the information provided. In addition, the users should be able to add their knowledge and understanding of the subject to gain the maximum benefit from this technology. 13 20 34

While ChatGPT 3 excels in theoretical knowledge, its limitations in clinical reasoning highlight the need for AI as a supplementary tool rather than a replacement. Our results, compared to similar studies in medical and dental AI education, show ChatGPT 4's improved accuracy but ongoing struggles with contextual understanding and challenges in dental education. ChatGPT 4 exhibited variable performance but overall demonstrated higher accuracy, achieving 64.4% in the Swiss Federal Licensing Examination in Dental Medicine. 35 This suggests an improved ability to handle a range of medical and dental exams with greater precision than its predecessor while enhancing its performance through specialized data sets and multimodal AI integration.

ChatGPT processes medical and dental information using a deep learning model trained on publicly available text data. However, it does not have direct access to proprietary dental or medical textbooks, peer-reviewed journal articles, or clinical case databases, which may limit its ability to provide fully accurate or context-specific responses. Bagde et al 36 reported that ChatGPT 4 showed strong potential for medical education, achieving 81% accuracy in licensing exams and outperforming ChatGPT 3.5 and many medical students. However, its inconsistent accuracy, variable performance, and differences in medical policies across countries limit its current suitability. While promising, further refinement is needed before integration into medical education.

Huang et al 37 reported that additionally biases in training data may influence its responses, as it relies on the most statistically relevant text patterns rather than true clinical reasoning. These factors contribute to its inconsistent accuracy compared to specialized AI models developed explicitly for medical education. Regarding ChatGPT's limitations in handling complex questions involving images, diagrams, and contextual cues, our study explicitly excluded image-based questions due to the model's inability to process visual data. Dental education, particularly subjects like dental anatomy, relies heavily on visual interpretation, which ChatGPT currently cannot perform. This limitation further supports our conclusion that while ChatGPT can serve as a supplementary learning tool, it cannot replace traditional educational methods or expert instruction. In a meta-analysis Jin et al 38 assessed ChatGPT 3.5 and ChatGPT 4 across four health licensing exams, finding ChatGPT 4 significantly more accurate. Performance was highest in pharmacy, followed by medicine, dentistry, and nursing. Future studies should expand question sets and explore advanced AI models for deeper insights into health care education and practice. Moreover, our study used the open-access version of ChatGPT 3.5, which has known limitations compared to ChatGPT 4. Future studies should explore the capabilities of more advanced AI models and assess how their improved contextual understanding impacts performance in medical and dental education. Furthermore, unlike our findings, Temiz and Güzel 39 reported that ChatGPT exhibited outstanding proficiency in the theoretical aspects of clinical sciences. However, true clinical competence extends beyond answering questions accurately, as its application in real-world practice remains constrained by its inability to replicate higher-order cognitive processes.

There are numerous papers written covering the potential use of ChatGPT for cheating in exams, but mostly limited to online exams. 11 40 In dental education, particularly in our settings, most of the assessments are physical, so unfair means are not a major challenge in face-to-face examinations. However, the authors agree with the perspective that the knowledge of AI should be integrated into the dental curriculum and faculty, and the students should be trained to use it ethically and responsibly. 41

Our study had several limitations. The study was limited to a single AI platform, ChatGPT, and tested a small segment of a dentistry course with a limited number of questions. Therefore, the results and implications may not be representative of other AI technologies, and further research is required to determine the generalizability of the findings. Our study findings are specific to ChatGPT and may not necessarily be representative of other AI technologies, and one of the primary limitations of our study is that it was conducted in a single dental institute, which restricts the generalizability of our findings to all dental students in the country. Additionally, our study utilized the open-access version of ChatGPT 3.5, which has limited capabilities compared to the more advanced ChatGPT 4. The differences in model capabilities could have influenced the test scores and explanation quality, as the responses generated by ChatGPT are highly dependent on the given prompts and version-specific features. Moreover, the educational system in Pakistan differs from that in Europe and North America. In many Western countries, dental students typically complete a bachelor's degree before entering dental school, equipping them with stronger foundational knowledge and critical thinking skills. In contrast, Pakistani dental students usually enter dental school directly after completing the twelfth grade. This structural difference in educational pathways makes direct comparisons between our cohort and other studies challenging. Further research, including multi-institutional studies with different AI models and advanced versions, is required to determine the generalizability of these findings and the true impact of AI-assisted learning in dental education.

The present study was limited to a single dental institute and used the open access of ChatGPT 3.5 with limited capabilities as compared to ChatGPT 4 with enhanced capabilities. The test and the explanation score might be affected due to the given prompt. The educational system in Pakistan is different from that in Europe and North America. In these countries, dental students typically complete a bachelor's degree before pursuing their dental degree. While in Pakistan, the dental students usually complete twelfth grade before admission into the dental school. Therefore, exact comparisons between our cohort and the other studies are not possible. ChatGPT and other AI programs are continuously evolving due to research, user feedback, and the availability of more data. Therefore, future studies using the same items may yield different results. Additionally, since this study only gathered the teacher's feedback on ChatGPT's explanation, future studies should also consider exploring the student's feedback on ChatGPT's explanations to provide valuable insights into its practical educational utility.

## Conclusion

Our study demonstrates that while ChatGPT possesses a foundational understanding of basic dental anatomy, sufficient to achieve a passing grade on an undergraduate examination, its performance exhibits limitations in accuracy and reliability. Therefore, it is crucial to emphasize that ChatGPT, and similar large language models, should not be considered a standalone e-learning or self-learning resource. Instead, these tools should be integrated as assistive technologies under the guidance of subject matter experts. To realize the full potential of AI in dental education, specific pathways for improvement must be pursued. Primarily, training AI models on meticulously curated, high-quality dental data sets is essential. This would involve incorporating diverse sources, including validated textbooks, peer-reviewed journals, clinical case studies, and expert-annotated radiographic images. Furthermore, implementing feedback loops with dental educators to refine the model's responses and explanations would enhance its accuracy and clinical relevance.

The future of AI in dental education lies in its ability to augment, not replace, traditional learning methods. By leveraging AI as a supplementary tool in the subjects like dental anatomy, we can enhance personalized learning and facilitate interactive learning by providing instant feedback and explanations for complex dental concepts. Future research should focus on evaluating a broader range of AI tools, exploring diverse dental subjects, and investigating the effectiveness of various AI-integrated pedagogical approaches.
