# Supplementary material for: Comparing ChatGPT and Dental Students' Performance in an Introduction to Dental Anatomy Examination: A Cross-Sectional Study
Source: Eur J Dent. 2025 May 13;20(1):287–94. doi: 10.1055/s-0045-1808254 (PMC12890407; doi:10.1055/s-0045-1808254)
Supplement: Supplementary file 1 — Supplementary Material [file 10-1055-s-0045-1808254-s2514046.pdf]

Multiple Choice Questions (MCQs) covering the introductory concepts of Dental Anatomy.

Sindh Institute of Oral Health Sciences

Department of Oral Biology

Dental Anatomy Test

2nd May 2023
Attempt all Questions Maximum Marks 25
Time: 25 minutes

1. The study of development morphology function and identity and relationship of the teeth in the human dentition is
   a. **Dental Anatomy**
   b. Dental Anthropology
   c. Dental Histology
   d. Forensic Odontology
2. The first permanent tooth erupts into the oral cavity around the age of
   a. 3 years
   b. **6 years**
   c. 12 years
   d. 14 years
3. The tissue with the least regenerative and repair potential is
   a. Bone
   b. Cementum
   c. Dentine
   d. **Enamel**
4. The part of the tooth visible in the oral cavity is:
   a. Anatomical crown
   b. **Clinical crown**
   c. Anatomical root
   d. Clinical root
5. How many teeth are present in one quadrant of a complete deciduous dentition?
   a. **5**
   b. 8
   c. 10
   d. 20
6. How many teeth are present in the maxillary arch of a complete permanent dentition?
   a. 8
   b. **16**
   c. 28
   d. 32
7. Which permanent tooth erupts into the space previously held by the primary second molar?
   a. First molar
   b. Second molar
   c. First premolar
   d. **Second premolar**
8. In the Universal Numbering System, what numbers are used to identify permanent maxillary lateral incisors?
   a. 1, 2
   b. **7, 10**
   c. 26, 23
   d. 25, 24
9. Saheer is observing an intact extracted molar tooth. which of the following junction is normally visible on this tooth?
   a. **Cementoenamel**
   b. Dentinoenamel
   c. Dentinocemental
   d. Dentogingival
10. A linear elevation on any surface of the tooth is a
    a. **Ridge**
    b. Cusp
    c. Tubercle
    d. Cingulum
11. A 5 years old boy came to the dental outpatient department complaining of severe pain in the lower left second molar. On clinical examination, the tooth is grossly carious. To further investigate this tooth, the dentist refers the patient to the radiology department for a periapical x-ray. This tooth in universal and federation dentaire international notation systems are designated as:
    a. 18, 37
    b. 31, 47
    c. **K, 75**
    d. T, 85
12. In a 15-year-old patient, mamelons indicate which of the following conditions?
    a. Amelogenesis Imperfecta
    b. Fenestration
    c. Fluorosis
    d. **Malocclusion**
13. In a five years old child with healthy dentition the total number of teeth present in the oral cavity are:
    a. 5
    b. 10
    c. 15
    d. **20**
14. In a five-year-old child the maxillary central incisors in the universal numbering system are designated as:
    a. A, B
    b. **E, F**
    c. 1, 2
    d. 8, 9
15. Which of the following anatomical landmarks on the tooth surface help in the cutting and grinding of food?
    a. Cervical line, Cingulum
    b. **Incisal ridges, Cusps**
    c. Mamelons, Grooves
    d. Marginal ridges, Fossa
16. Which of the following term is used for the surfaces of the teeth facing towards adjoining teeth in the same dental arch?
    a. **Proximal**
    b. Mesial
    c. Distal
    d. Marginal ridge

 © 2025. The Author(s).

17. The term describes the small rounded protuberances on the cutting surfaces of newly erupted incisors.
    a. Tubercle
    b. Cingulum
    c. Fossa
    d. **Mamelon**
18. Which of the following malformation is common in the permanent maxillary lateral incisor?
    a. shovel-shaped
    b. **Peg shape**
    c. Hypoplasia
    d. Screwdriver shape
19. Which of the following anatomical landmark is present only on the anterior teeth?
    a. Fossa
    b. Pit
    c. Ridge
    d. **Cingulum**
20. The first deciduous tooth that emerges into the oral cavity is:
    a. **Mandibular central**
    b. Mandibular lateral
    c. Maxillary central
    d. Maxillary lateral
21. The tissue which covers the root of the tooth.
    a. **Cementum**
    b. Periodontal ligaments
    c. Bone
    d. Dentin
22. Which of the following tissue of the tooth is deposited in response to overeruption?
    a. Enamel
    b. Dentin
    c. **Cementum**
    d. Pulp
23. The mixed dentition period begins which the eruption of which of the following permanent tooth?
    a. Mandibular central incisors
    b. **Mandibular first molar**
    c. Maxillary central incisor
    d. Maxillary first molar
24. When the permanent mandibular first molar emerges into the oral cavity the tooth present mesial to the tooth is:
    a. Deciduous first molar
    b. **Deciduous second molar**
    c. Permanent first premolar
    d. Permanent second premolar
25. The prenatal dental age estimation is commonly based on which of the following method?
    a. Teeth emergence through the gingiva
    b. Attrition and wear of teeth
    c. **Dissected fetal material**
    d. Radiological data

Supplementary Table A1 Hypothesis test summary

	Null hypothesis	Test	Significance	Decision
1	The distribution of average is normal with mean 4.72 and standard deviation 4.48628	One-sample Kolmogorov–Smirnov test	0.004[a]	Reject the null hypothesis
Asymptotic significances are displayed. The significance level is 0.050				

[a]Lilliefors corrected.

Supplementary Table A2 Tests of normality

	Kolmogorov–Smirnov[a]			Shapiro–Wilk		
	Statistic	df	Significance	Statistic	df	Significance
Average	0.217	25	0.004	0.769	25	0.000

Abbreviation: df, degree of freedom.
[a]Lilliefors significance correction.

Supplementary Table A3 Correlations between the two independent experts J1 and J2

			J1	J2
Spearman's rho	J1	Correlation coefficient	1.000	0.942[a]
		Significance (two-tailed)		0.000
		N	25	25
	J2	Correlation coefficient	0.942[a]	1.000
		Significance (two-tailed)	0.000	
		N	25	25

[a]Correlation is significant at the 0.01 level (two-tailed).

 © 2025. The Author(s).

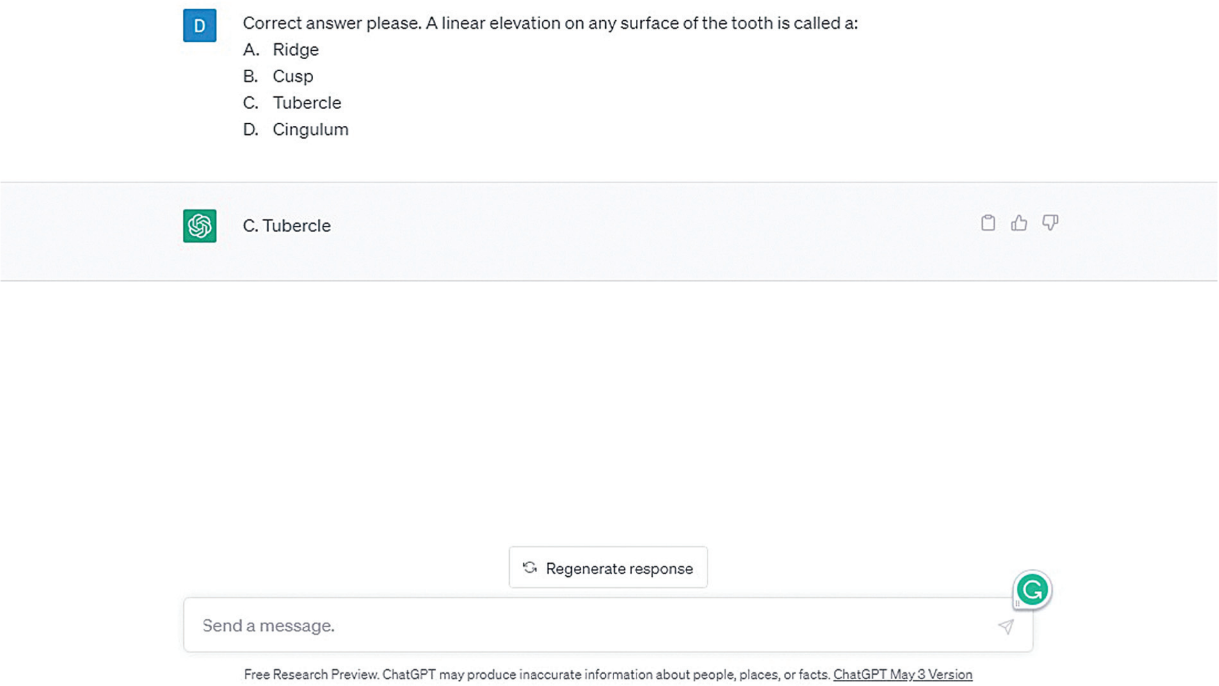

Supplementary Fig. A1 Screenshot of the ChatGPT incorrect answer with no explanation.

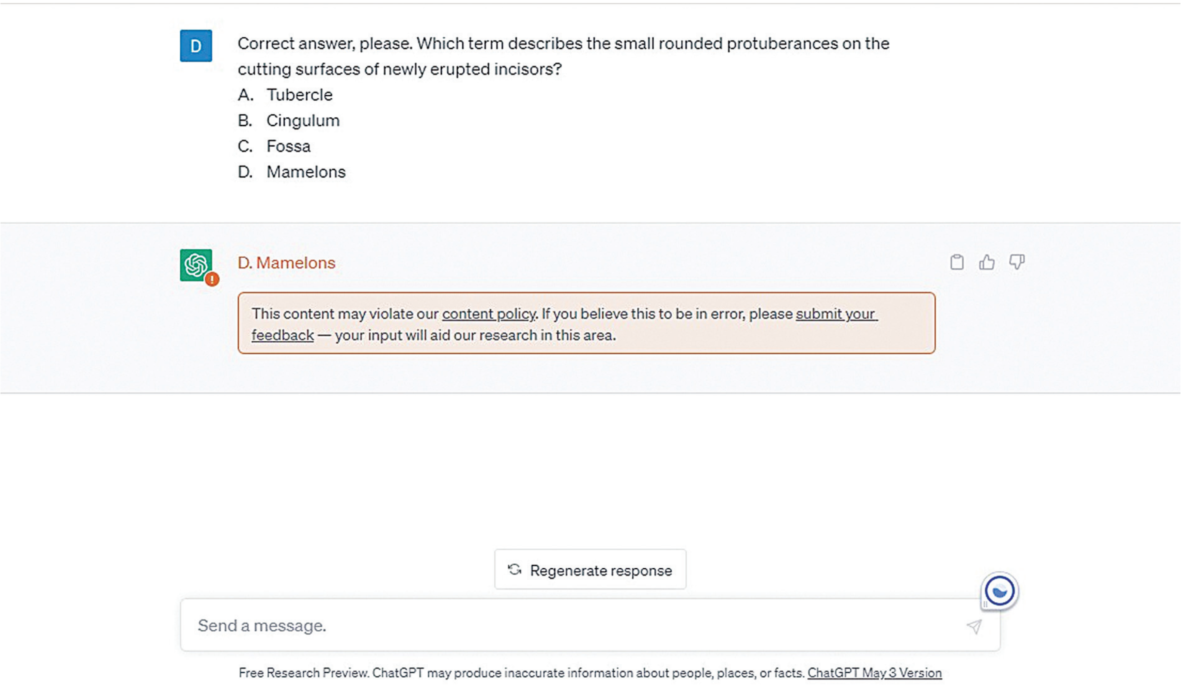

Supplementary Fig. A2 Screenshot of the ChatGPT correct answer with error notification.

 © 2025. The Author(s).

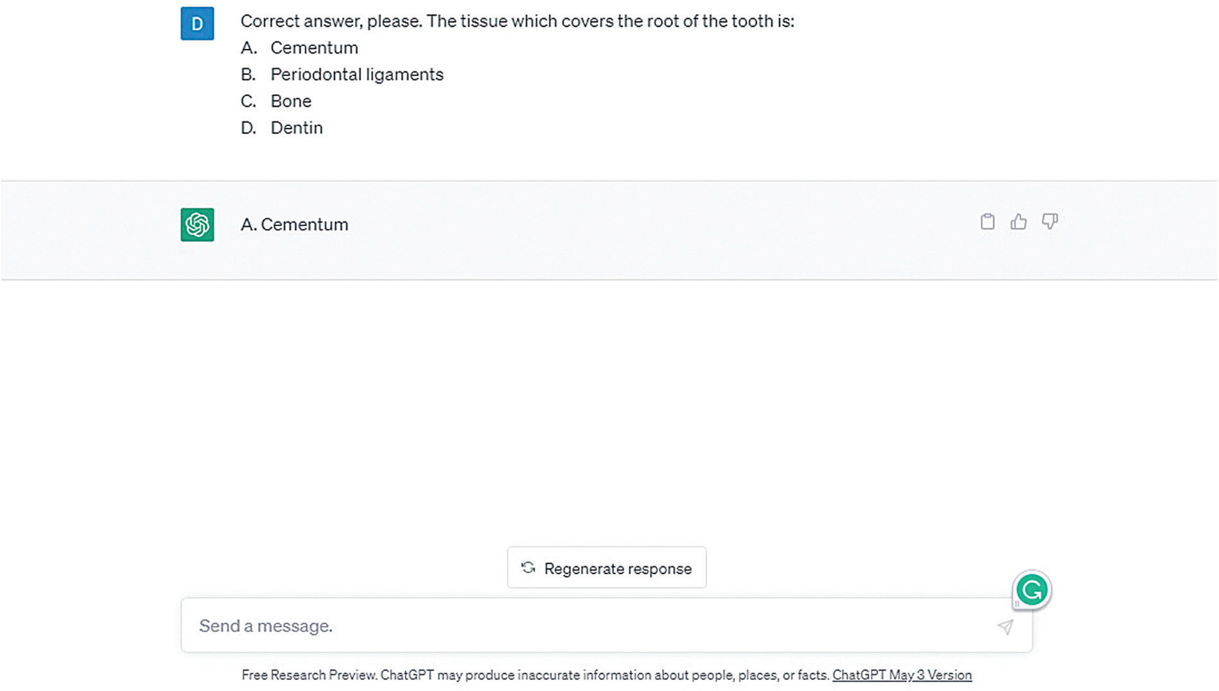

Supplementary Fig. A3 Screenshot of the ChatGPT correct answer with no explanation.

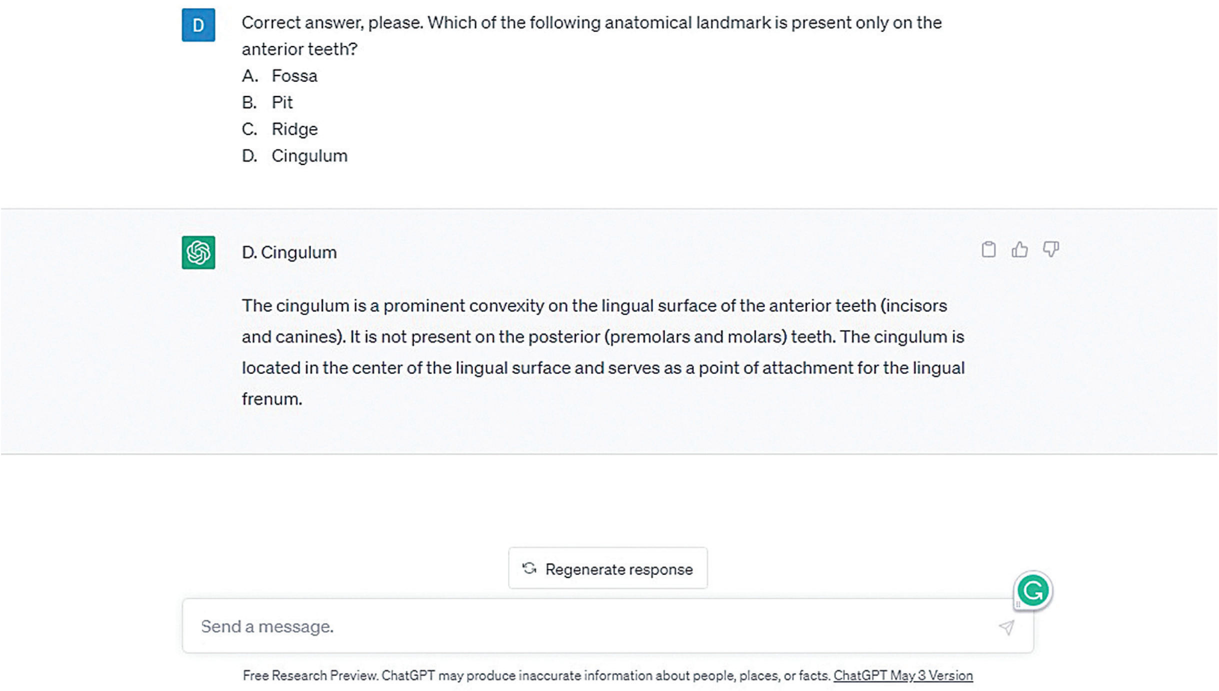

Supplementary Fig. A4 Screenshot of the ChatGPT correct answer with partially correct explanation.

 © 2025. The Author(s).
